# Unexplained Acute Total Loss of Vision After Primary Scleral Buckle Surgery

**DOI:** 10.1177/24741264241260483

**Published:** 2024-06-22

**Authors:** Greg Budoff, Alexander B. Dillon, Adrian Au, Allan E. Krieger, Steven D. Schwartz

**Affiliations:** 1 Retina Division, Jules Stein Eye Institution, UCLA, Los Angeles, CA, USA; 2 Retina Consultants, Hartford, CT, USA; 3 East Bay Retina Consultants, Oakland, CA, USA

**Keywords:** scleral buckle, retinal detachment surgery, blindness, ocular ischemia, complications

## Abstract

**Purpose:** To present 2 cases of acute total loss of vision after scleral buckle surgery for rhegmatogenous retinal detachment. **Methods:** A retrospective chart review of 2 cases and an analysis of the literature were performed. **Results:** An 18-year-old woman and a 67-year-old woman suffered complete loss of vision in their operative eye after primary scleral buckle surgery with encircling bands. **Conclusions:** Profound ocular ischemia resulting in total acute vision loss is a rare and devastating outcome of primary scleral buckle procedures and may be caused by strangulation of the eye with an encircling band. Attention paid to the key tenets of this often successful and useful surgical technique may lower the risk for this complication.

## Introduction

The scleral buckle procedure is a definitive method of repairing rhegmatogenous retinal detachment (RRD) with high functional and anatomic success rates.^1,2^ However, decreased use of this technique over recent decades is well documented^3^ because of the potential complications, including failure to achieve retinal reattachment, choroidal detachment, intraocular hemorrhage, strabismus, myopic shift, and infection. Ocular surgery, including scleral buckling, rarely results in morbid, unexpected, and difficult-to-explain outcomes, such as retinal artery occlusion (RAO).^4-10^ We present 2 patients with acute postoperative total loss of vision in the operated eye after a scleral buckle procedure for RRD who were referred to our institution.

## Case Reports

### Case 1

An 18-year-old woman without a significant medical history was emergently referred to our center 1 day after scleral buckle surgery with an encircling 41 band using scleral belt loops and Watzke sleeve to treat a macula-on RD in the left eye. The procedure was performed under general anesthesia and ended with a sub-Tenon anesthetic injection of 2 mL of a 50:50 mixture of 2% lidocaine and 0.5% bupivacaine. No untoward intraoperative systemic hypotensive events or cardiac arrhythmias were included in the anesthesia records. Per the operative report, indirect ophthalmoscopy before closure showed retinal vascular perfusion and a normotensive intraocular pressure (IOP) on palpation before and after anterior chamber paracentesis was performed. No external drainage of subretinal fluid (SRF) was done.

**Figure 1.**
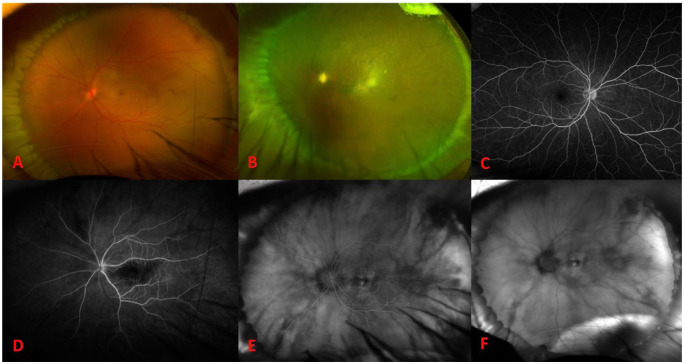
(A) Widefield fundus photograph of the involved eye on postoperative day 1 shows a cherry-red spot. Widefield fundus photograph of the involved eye at postoperative month 6 shows sclerotic vessels and a pale optic nerve. (C) Normal fluorescein angiography (FA) of the fellow eye on postoperative day 1. FA of the involved eye on postoperative day 1 shows early perfusion of the retinal vessels and patchy choroidal filling with late macular and choroidal leakage at (D) 0:23, (E) 3:05, and (F) 11:00.

On presentation to our institution, the VA in the left eye was no light perception (NLP) with an afferent pupillary defect and the IOP was 19 mm Hg. The VA in the fellow eye was 20/20. On examination, the retina was flat with retinal whitening, a cherry-red spot, and a high anterior encircling scleral buckle lending a view to the ora serrata without scleral depression (Figure 1A). Fluorescein angiography (FA) showed retinal arteriolar reperfusion with patchy choroidal filling as well as late macular and peripheral choroidal leakage (Figure 1, D–F).

The patient was admitted to the hospital for an expedited stroke, vasculitis, and hypercoagulable workup that was largely unremarkable aside from indeterminately elevated anticardiolipin immunoglobulin M (14.4 mg/dL; normal, <12.5 mg/dL) and slight leukocytosis (13.4 × 10^9^ cells/L; normal, <10 × 10^9^ cells/L) that was presumed to be reactive secondary to her postsurgical status.^4^ Carotid studies, an echocardiogram, and neuroimaging were normal.

**Figure 2.**
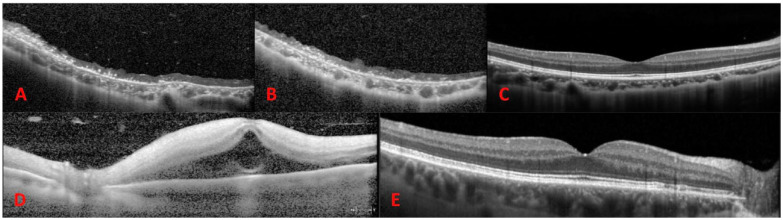
(A) Baseline and (B) 6-month postoperative optical coherence tomography (OCT) images of the involved eye in Case 1 shows inner and outer retinal atrophy. (C) The fellow eye is normal. (D) OCT on postoperative day 4 of the involved eye of Case 2 shows inner and outer retinal hyperreflectivity with some residual subretinal fluid. (E) The fellow eye shows rare drusenoid changes to the nasal macula but is otherwise unremarkable.

Despite 6 sessions of inpatient hyperbaric oxygen treatment over 3 days, the VA in the patient’s operative eye did not recover. Optical coherence tomography (OCT) of the macula showed diffuse thinning and disorganization of the inner retinal layer and outer retinal layer (Figure 2, A–B). At the 6-month follow-up, the patient’s examination was otherwise significant for a nascent cataract and sclerotic retinal vasculature (Figure 1B). The fellow eye was normal (Figure 1, C and Figure 2C).

### Case 2

A 67-year-old woman with a medical history of stage 1 uterine cancer status a total abdominal hysterectomy and bilateral salpingo-oophorectomy was referred for acute total loss of vision after repair of a macula-involving RRD with scleral buckling in the left eye. Her medical history also included well-controlled iron deficiency anemia, hormone replacement therapy, and osteoporosis, for which she took denosumab.

The scleral buckle procedure began with a retrobulbar anesthetic injection of 5 mL of a 50:50 mixture of 2% lidocaine and 0.75% bupivacaine. With the patient under monitored anesthesia care (MAC), the surgeon used an encircling 42 band with imbricating intrascleral 5-0 nylon mattress sutures in 4 quadrants secured with a 70 sleeve. Anesthesia records were reviewed, and no untoward systemic hypotensive events or cardiac arrhythmias occurred during the procedure. Anterior chamber paracentesis was performed twice per the operative note, after which the IOP was documented as normal on palpation. External SRF drainage was not performed. An intravenous infusion of acetazolamide 500 mg was administered at the end of the case.

Although indirect ophthalmoscopy was reportedly performed at least twice intraoperatively, the perfusion status of the retinal vasculature and optic nerve head was not documented. On postoperative day 1, the patient’s vision was NLP; anterior chamber paracentesis was performed to further lower the already normal IOP.

**Figure 3.**
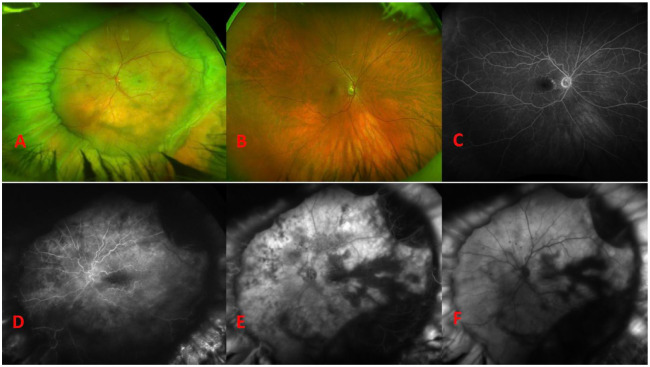
(A) Widefield fundus photograph of the involved eye on postoperative day 4 shows a high and broad scleral buckle with a cherry-red spot and midperipheral intraretinal hemorrhages. The fellow eye shows some macular drusen (B) that stain (C) on fluorescein angiography (FA). FA of the involved eye on postoperative day 4 shows perfusion of the retinal vessels with patchy choroidal filling and later choroidal leakage at (D) 0:25, (E) 1:04, and (F) 10:15.

The patient was emergently referred to our tertiary care center on postoperative day 4. At that time, the VA in the left eye remained NLP with an afferent pupillary defect and an IOP of 8 mm Hg. The anterior segment examination showed expected postoperative findings. Notably absent were signs of inflammation or anterior segment ischemia, such as cell, flare, or engorgement of the iris vessels. The anterior chamber depth appeared normal. A fundus examination showed a notably high, broad scleral buckle with multiple overlying radial retinal folds (Figure 3A). The retina was grossly flat aside from a small amount of residual SRF just posterior to the scleral buckle. There was a macular cherry-red spot and scattered midperipheral intraretinal hemorrhages. FA showed retinal artery reperfusion, delayed, patchy choroidal filling, and late choroidal leakage (Figure 3, D–F). OCT showed hyperreflectivity of all layers of the retina with some residual SRF (Figure 2D). The fellow eye showed few staining macular drusen with normal retinal and choroidal vasculature (Figure 2, E and 3C). The patient was observed without further intervention.

## Conclusions

Complete loss of LP immediately after scleral buckling occurred in 2 patients referred to our institution, both of whom showed signs suggestive of ophthalmic artery occlusion. NLP vision, outer retinal involvement on OCT, and late choroidal leakage on FA are signs suggestive of ophthalmic artery occlusion, whereas in general, central RAO (CRAO) results in hand motions or better VA, typically shows only inner retinal involvement, and is not usually seen without a vasculitic etiology.

Several cases of CRAO after ocular surgical procedures have been reported.^4-11^ To our knowledge, no other case of an ophthalmic arterial occlusion resulting in total loss of vision has been reported after scleral buckling. A discussion of the pathophysiology of these cases and the associated differential diagnosis may be informative.

Numerous cases reports have described CRAO occurring on the first postoperative day after an ocular surgery with preoperative retrobulbar anesthesia.^6,7^ The mechanism is presumed to be direct injury to the central retinal artery from the retrobulbar needle or compression of the central retinal artery from a retrobulbar hemorrhage. The surgery in Case 1 was performed under general anesthesia with a small sub-Tenon injection via a cannula, obviating the risk for direct retinal artery trauma. The surgery in Case 2 was performed under MAC with a retrobulbar block during which direct or indirect local anesthetic injection trauma may have occurred.

In addition to the risk for globe perforation, retrobulbar anesthesia carries the risk for toxicity. Six cases of RAO after retrobulbar injection of anesthetic agents containing preservatives have been reported.^11^ An important caveat of this mechanism is that the toxicity takes time to develop, and the occlusion does not occur until at least postoperative day 2, although it can present as late as day 14. Both instances of arterial occlusion in our series occurred on postoperative day 1, with no evidence of posterior segment globe perforation. Thus, this is unlikely to be the etiology of vision loss in our patients.

Encircling scleral buckles have been shown to reduce ocular blood flow.^12-15^ Intraoperative systemic hypotension during scleral buckling, while not reflected in our patients’ records, has been associated with ophthalmic artery hypoperfusion.^5^ During scleral buckling, globe proptosis can occur, putting stress on and reducing blood flow through the ophthalmic artery. Elevated IOP can also reduce ocular perfusion, making frequent monitoring of the central retinal artery perfusion and IOP during scleral buckling procedures imperative. IOP-lowering maneuvers, such as anterior chamber paracentesis, external drainage of SRF, or loosening of the scleral buckle or sutures, must be performed during the scleral buckling procedure if the central retinal artery is not perfused. According to the operative reports, central retinal artery perfusion was confirmed at the end of Case 1 but not Case 2. Although the IOP was physiologic on postoperative day 1 in both cases, it is possible that it was transiently elevated postoperatively, jeopardizing arterial perfusion.

Classically, the most common etiology of a RAO is thromboembolic secondary to a cardiac or carotid embolic source. Although not impossible, this would be extremely unlikely in the immediate postoperative course, in particular in a young adult like our first patient. Furthermore, corresponding risk factors were ruled out in this case during the inpatient workup. Ophthalmic or RAO after cosmetic facial injection of autologous fat, hyaluronic acid, and collagen (“dermal fillers”) has been described.^16^ The mechanism is presumed to be retrograde flow of the injected substance to the ophthalmic or retinal arterial system caused by the high force of injection. In these reports, the filler is typically visualized intravascularly on fundus examination. In our patients, no iatrogenic embolic source or directly visualizable embolism was identified.

Vasculitis and hypercoagulable states are known to be associated with CRAO in young people^17^; however, these conditions are not known to cause ophthalmic artery occlusions. Vasculitis is associated with a characteristic retinal vascular leakage on FA not seen in our cases. Likewise, the vasculitis workup for our first patient was largely unremarkable.

Scleral buckling is an effective procedure to repair an RD. Some evidence indicates that the anatomic and functional outcomes after scleral buckling may be better than those after pars plana vitrectomy (PPV).^18,19^ Most RDs can be repaired successfully with more than 1 procedural technique. There are, however, a few instances in which scleral buckling may be indicated over PPV, including in cases of RDs without a posterior vitreous detachment, in young phakic patients with a clear lens, or in patients who have to ascend in altitude in the early postoperative course. It is thus essential that a vitreoretinal surgeon be comfortable with scleral buckling and take the necessary precautions to avoid the potential complications associated with this surgery.

To our knowledge, no other cases of presumed ophthalmic artery occlusion and complete loss of vision immediately after an encircling scleral buckling procedure for repair of an RRD have been reported. A discussion of the pathophysiology of these cases and the associated differential diagnosis may be educational.
